# The Imperative Role of Xpert® Mycobacterium Tuberculosis Complex/Resistance to Rifampin (MTB/RIF) in Rapid Diagnosis of Pulmonary and Extrapulmonary Tuberculosis

**DOI:** 10.7759/cureus.76706

**Published:** 2024-12-31

**Authors:** Santhiya Ramachandran, Pajanivel R

**Affiliations:** 1 Pulmonary Medicine, Mahatma Gandhi Medical College and Research Institute, Sri Balaji Vidyapeeth (Deemed to be University), Puducherry, IND

**Keywords:** gene xpert mtb/rif assay, multi-drug resistant tb -mdr, rifampicin resistance, sputum smear negative, tuberculosis testing

## Abstract

Background

The Xpert® mycobacterium tuberculosis complex/resistance to rifampin (MTB/RIF) test is widely recognized for its ability to provide rapid and accurate results for diagnosing tuberculosis (TB) in both pulmonary and extrapulmonary samples. Its utility is especially critical in smear-negative and paucibacillary cases where traditional diagnostic methods often fail. This study aimed to evaluate the role of Xpert® MTB/RIF in diagnosing TB in various pulmonary and extrapulmonary samples and TB in smear-negative pulmonary samples.

Methods

This retrospective study analyzed records from 2017 to 2021, focusing on presumptive TB patients who underwent Xpert® MTB/RIF testing at a tertiary care hospital. Data from 1174 samples (671 pulmonary and 503 extrapulmonary) were included. The Xpert® MTB/RIF reports were assessed for *Mycobacterium tuberculosis* (MTB) detection rates across different sample types.

Results

Of the pulmonary samples, MTB detection was highest in sputum samples, 122 (48.41%), followed by bronchial washing samples, 49 (16.33%). In smear-negative pulmonary cases, Xpert® MTB/RIF detected MTB in 72 (16.55%) samples, demonstrating its utility in paucibacillary cases. The test also proved valuable in diagnosing extrapulmonary TB, where symptoms are often atypical.

Conclusion

Xpert® MTB/RIF is a reliable tool for the rapid and accurate diagnosis of pulmonary and extrapulmonary TB, including smear-negative and paucibacillary cases. Its application can significantly enhance the timely diagnosis and treatment of TB, especially in resource-limited settings.

## Introduction

Tuberculosis (TB) continues to pose a major public health challenge globally, with India bearing a significant proportion of the burden. As per the World Health Organization (WHO), approximately 10 million people worldwide developed TB in 2018, with India accounting for 2.69 million cases, making it one of the eight countries responsible for two-thirds of the global TB burden [1]. This alarming statistic underscores the need for timely and accurate diagnosis, as early detection is crucial for reducing disease transmission, improving patient outcomes, and achieving global TB elimination goals​.

Conventional diagnostic methods for TB, such as sputum microscopy and culture techniques, have notable limitations. While sputum microscopy is easy to perform, its sensitivity is suboptimal, especially in smear-negative and paucibacillary TB cases [2]. Culture methods, although highly sensitive, are time-consuming, with results often taking weeks to obtain. These limitations delay treatment initiation and hinder TB control efforts. To address these gaps, the WHO endorsed nucleic acid amplification tests (NAATs) in 2010, with the Xpert® mycobacterium tuberculosis complex/resistance to rifampin (MTB/RIF) test emerging as a game-changer in the field of TB diagnostics [3].

The Xpert® MTB/RIF test is a cartridge-based, fully automated molecular diagnostic tool that employs real-time polymerase chain reaction (PCR) technology to detect *Mycobacterium tuberculosis* (MTB) and rifampicin resistance within two hours. This rapid turnaround time, combined with its ability to process both pulmonary and extrapulmonary samples, makes Xpert® MTB/RIF a valuable first-line diagnostic tool. It has been shown to have a pooled sensitivity of 88% and specificity of 99% for detecting *TB bacilli*, comparable to liquid culture methods. Furthermore, its sensitivity in detecting smear-negative, culture-positive TB cases is reported at 68%, demonstrating its utility in challenging diagnostic scenarios. When used to detect rifampicin resistance, Xpert® MTB/RIF has a sensitivity of 95% and specificity of 98% compared to phenotypic reference standards. When used to diagnose extrapulmonary TB, Xpert® MTB/RIF has a pooled sensitivity of 84.9% for lymph node/aspirate, 83.8% for gastric aspirate, 79.5% for CSF, 43.7% for pleural fluid, and 81.2% for other tissue specimens [4].

Several studies have validated the efficacy of Xpert® MTB/RIF in diverse clinical contexts [5-10]. For pulmonary TB, the test demonstrates a high diagnostic yield in sputum and bronchial washing samples, even in smear-negative cases. In extrapulmonary TB, its sensitivity varies by sample type, with higher detection rates in lymph node aspirates and pus compared to fluids like pleural or pericardial fluid. These findings emphasize the versatility of Xpert® MTB/RIF in diagnosing both pulmonary and extrapulmonary TB, including cases with atypical presentations or low bacterial loads.

Given the increasing TB burden in India, innovative diagnostic tools like Xpert® MTB/RIF play an imperative role in controlling the epidemic. By enabling early and accurate diagnosis, the test not only improves treatment outcomes but also reduces disease transmission and the emergence of drug-resistant strains. This study evaluates the performance of Xpert® MTB/RIF in detecting TB across pulmonary and extrapulmonary samples, with a special focus on smear-negative cases. Additionally, the study aims to compare the performance of sputum direct microscopy with Xpert® MTB/RIF in sputum and bronchial wash samples and to correlate patients’ symptoms with the results from sputum direct microscopy and Xpert® MTB/RIF for sputum samples. Lastly, the study seeks to determine the prevalence of drug-resistant TB within the study population.

## Materials and methods

Study design and setting

This was a retrospective, record-based study conducted at a tertiary care hospital in Pondicherry, India, spanning five years (2017-2021). The study aimed to analyze the diagnostic performance of Xpert® MTB/RIF in detecting TB across various pulmonary and extrapulmonary samples. Ethical clearance for the study was obtained from the Institutional Ethics Committee, MGMCRI (MGMCRI/IRC/38/2020/04/IHEC/148), ensuring adherence to ethical research standards.

Study variables

The study variables were classified as follows:

The study's independent variables encompassed demographic, clinical, and specimen-related factors. Age, a quantitative variable, was analyzed as mean ± standard deviation to assess its influence on TB detection rates. Gender, a qualitative variable, was categorized into male, female, or transgender groups to identify any gender-based diagnostic variations. Clinical symptoms such as cough, weight loss, fever, and asymptomatic presentations were evaluated to correlate symptomatology with TB detection. Specimen type was classified into pulmonary and extrapulmonary to assess the diagnostic performance of Xpert® MTB/RIF across diverse sample origins. Additionally, the prior history of antitubercular treatment intake was included to evaluate its impact on diagnostic outcomes.

The dependent variables focused on diagnostic results. These included Xpert® MTB/RIF outcomes, specifically MTB detection and rifampicin resistance, highlighting the assay’s utility in identifying *Mycobacterium tuberculosis* and drug resistance patterns. Drug sensitivity or resistance outcomes were also examined to provide insights into the resistance profiles within the study population, essential for tailoring appropriate treatment regimens. This comprehensive set of variables ensured a robust analysis of the diagnostic and clinical implications of Xpert® MTB/RIF in TB management.

Sample size calculation

The sample size was calculated based on a previous study by Mathur RB et al. [9], which reported a prevalence of 40% for MTB detection using Xpert® MTB/RIF. Using a 95% confidence interval and 4% precision, the required sample size was determined to be 633. However, the final study analyzed 1174 samples, ensuring robustness.

Data collection procedure

**Figure 1:**
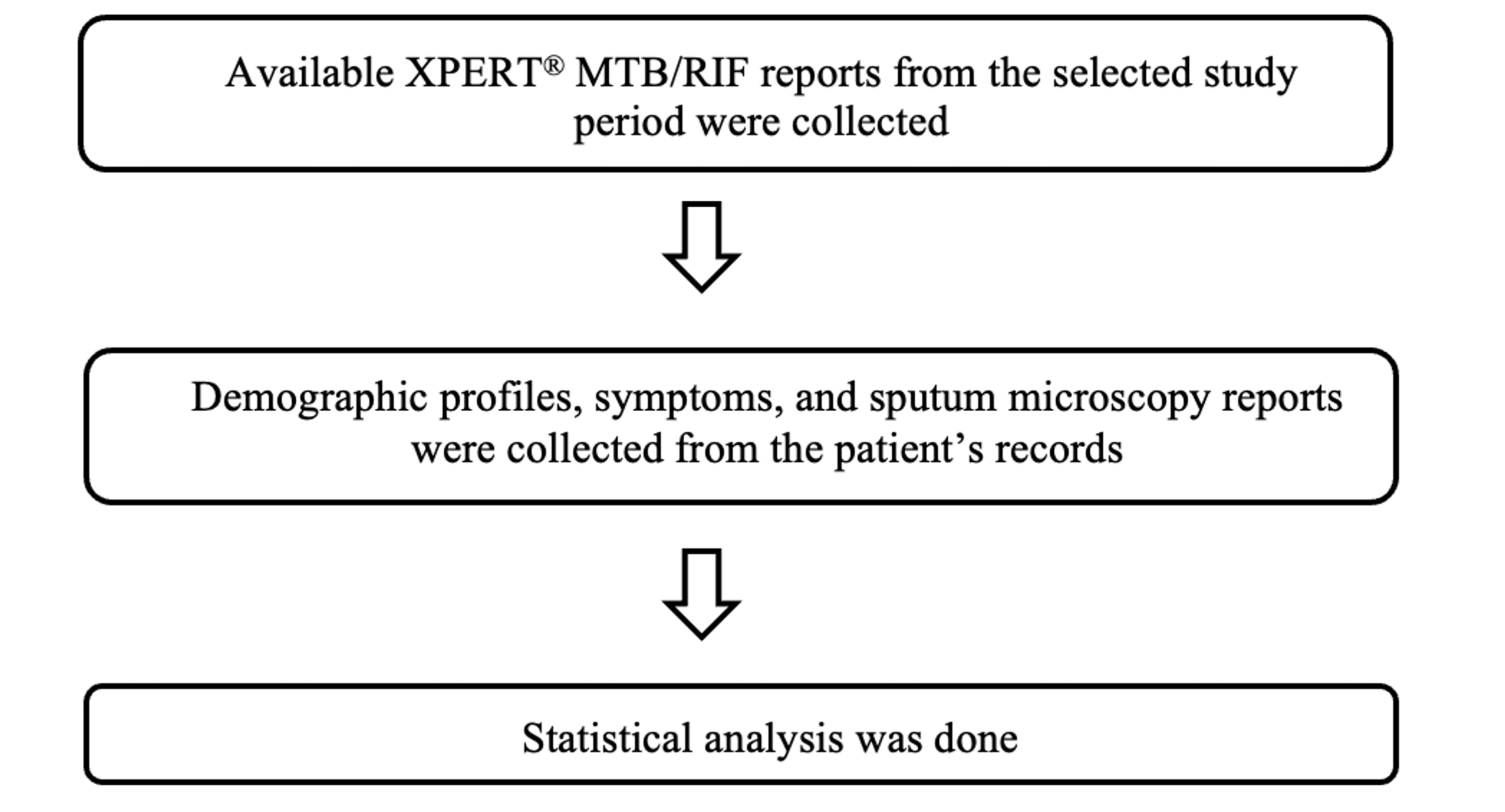
Sequence of events in the study

Data were collected from hospital records of patients who underwent Xpert® MTB/RIF testing during the study period. The process included the following steps as given in Figure 1.

Sample selection: universal sampling was employed to include all presumptive TB cases for which Xpert® MTB/RIF testing was conducted.

Inclusion and exclusion criteria

Inclusion: patients with complete microbiological workups, including Xpert® MTB/RIF results, were included.

Exclusion: patients with incomplete diagnostic records or missing Xpert® MTB/RIF results were excluded.

Variables extracted: data on demographic characteristics (age, gender), clinical symptoms, HIV status, history of antitubercular treatment (ATT), and type of specimen (pulmonary or extrapulmonary) were retrieved. Additionally, Xpert® MTB/RIF results, including MTB detection and rifampicin resistance, were recorded.

Categorization of samples: samples were classified as pulmonary (e.g., sputum, bronchial wash) or extrapulmonary (e.g., lymph node aspirates, pleural fluid) based on their origin.

Laboratory testing and analysis

All specimens were processed using Xpert® MTB/RIF, a cartridge-based nucleic acid amplification test (CBNAAT). This automated molecular test detects MTB and rifampicin resistance in approximately two hours, using real-time polymerase chain reaction (RT-PCR) technology. The pulmonary samples included sputum, bronchial washings, and gastric aspirates, while extrapulmonary samples included lymph node aspirates, pleural fluid, and bone pus. The results were categorized as MTB detected, not detected, or indeterminate, with rifampicin sensitivity or resistance noted.

Statistical analysis

The data were entered into Microsoft Excel (Redmond, USA) and analyzed using IBM Corp. Released 2012. IBM SPSS Statistics for Windows, Version 21.0. Armonk, NY: IBM Corp. Both descriptive and inferential statistical methods were applied. Continuous variables like age were summarized using mean and standard deviation. Categorical variables like gender, specimen type, and MTB detection rates were presented as frequencies and percentages. The chi-square or Fisher’s exact test was used to assess the association between qualitative variables, such as specimen type and MTB detection rates. A p-value <0.05 was considered statistically significant.

## Results

Demographic and clinical characteristics

A total of 1174 samples from presumptive tuberculosis (TB) patients were included in the study, of which 671 (57.2%) were pulmonary samples and 503 (42.8%) were extrapulmonary samples (Table 1). Among the patients, males constituted 780 (66.4), while females accounted for 393 (33.4). The mean age of the patients was 38.6 ± 12.4 years, with a significant proportion aged between 31 and 60 years, reflecting the working-age population, which is often at higher risk for TB due to increased exposure and occupational factors. Clinical symptoms such as cough, fever, and weight loss were observed in 526 (78.3%), 323 (65.2%), and 370 (54.6%) of patients, respectively. Among the samples, 113 (17%) in the pulmonary group and 49 (10%) in the extrapulmonary group belong to individuals with a history of anti-tubercular drugs.

**Table 1:** Characteristics between pulmonary and extrapulmonary samples collected (n=1174) H/o ATT: History of any anti-tuberculosis treatment

Variables	Pulmonary sample (n=671) N (%)	Extra-pulmonary sample (n=503) N (%)
Age group(years)		
<15	89 (13.26)	49 (9.74)
16-30	63 (9.39)	118 (23.46)
31-60	372 (55.44)	266 (52.88)
>60	147 (21.91)	70 (13.92)
Gender		
Male	470 (70.04)	310 (61.63)
Female	201 (29.96)	192 (38.17)
Transgender	0 (0)	1 (0.2)
HIV status		
Unknown	190 (28.32)	203 (40.36)
Positive	10 (1.49)	2 (0.4)
Negative	471 (70.19)	298 (59.24)
H/o ATT		
Unknown	32 (5)	201 (40)
Present	113 (17)	49 (10)
Absent	526 (78)	253 (50)

Among the 671 pulmonary samples analyzed, MTB detection was highest in sputum samples, with a positivity rate seen in 122 (48.41%), followed by bronchial wash samples at 49 (16.33%). These findings demonstrate the effectiveness of Xpert® MTB/RIF in detecting MTB in respiratory specimens, particularly in sputum samples, which remain the primary diagnostic material for pulmonary TB (Table 2).

**Table 2:** MTB detection by CB-NAAT in pulmonary samples (n=671) CB-NAAT: Cartridge based nucleic acid amplification test

SPECIMEN	Detected N (%)	Not detected N (%)
Bronchial washing	49 (16.33)	251 (83.67)
ET aspirate	1 (6.25)	15 (93.75)
Gastric aspirate	1 (1.2)	82 (98.8)
Lung tissue	0 (0)	20 (100)
Sputum	122 (48.41)	130 (51.59)

For the 503 extrapulmonary samples, lymph node aspirates yielded the highest detection rate at eight (53.33%), followed by bone pus/aspirate (47.37%), and the least MTB detection was with breast, synovial, and pericardial tissue (0%) as in Table 3. The detection rates in extrapulmonary samples emphasize the versatility of Xpert® MTB/RIF in diagnosing TB in specimens with atypical presentations and low bacterial loads. Overall, MTB was detected in 453 (38.6%) of the total samples, highlighting the significant diagnostic yield of Xpert® MTB/RIF in diverse clinical settings.

**Table 3:** MTB detection by CBNAAT in extrapulmonary samples (n=503) CSF: Cerebrospinal fluid, LN: Lymph node, CB-NAAT: Cartridge based nucleic acid amplification test

	Detected N (%)	Not detected N (%)
ASCITIC FLUID	1 (3.33)	29 (96.67)
BONE BIOPSY	4 (11.43)	31 (88.57)
BONE PUS/ASPIRATE	18 (47.37)	20 (52.63)
BRAIN TISSUE	2 (28.57)	5 (71.43)
BREAST TISSUE	0 (0)	7 (100)
CSF	6 (11.32)	47 (88.68)
EMPYEMA	4 (50)	4 (50)
LN	22 (32.35)	46 (67.65)
LN Pus	8 (53.33)	7 (46.67)
OTHER BIOPSY	4 (9.52)	38 (90.48)
OTHER PUS	14 (38.89)	22 (61.11)
PERICARDIAL FLUID	1 (14.29)	6 (85.71)
PERICARDIUM TISSUE	0 (0)	2 (100)
PLEURAL FLUID	9 (13.24)	59 (86.76)
PLEURAL PEEL	19 (31.15)	42 (68.85)
SYNOVIAL FLUID	1 (4.55)	21 (95.45)
SYNOVIUM	0 (0)	4 (100)

The Xpert® MTB/RIF test demonstrated a detection rate of 72 (16.55%) among 435 smear-negative pulmonary samples, underscoring its effectiveness in diagnosing paucibacillary tuberculosis (TB) cases. This result was statistically significant (p = 0.03), highlighting the test’s sensitivity in detecting *Mycobacterium tuberculosis* (MTB), where traditional smear microscopy often fails due to low bacterial loads. Smear-negative cases, which pose a diagnostic challenge and are often associated with delayed treatment, can benefit greatly from the rapid and accurate detection offered by Xpert® MTB/RIF. 

In our study, the association between MTB detection and symptomatology among pulmonary samples was analyzed, as shown in Table 4. Among the 671 pulmonary samples, 233 (34.7%) patients presented with cough accompanied by sputum or hemoptysis, of which MTB was detected in 70 cases (30.04%). A larger group of 410 (61.1%) patients exhibited symptoms such as dry cough, fever, or weight loss, with MTB detection observed in 100 cases (24.39%). Notably, 28 (4.2%) patients were asymptomatic, and MTB was detected in only three of these cases (10.71%). The statistical analysis revealed a significant association between MTB detection and symptomatology (p = 0.001).

**Table 4:** Association of MTB detection with symptomatology among the study participants (n=671) Chi-square/Fisher’s exact test

	MTB		Total	p-value
Symptoms	Detected (n, %)	Not detected (n, %)		0.001
Cough with sputum hemoptysis	70 (30.04)	163 (69.96)	233	
Dry cough/fever/weight loss	100 (24.39)	310 (75.61)	410	
Asymptomatic	3 (10.71)	25 (89.29)	28	
Total	173 (25.78)	498 (74.42)	671	

The distribution of sensitivity, resistance, and indeterminate results for rifampicin among pulmonary and extrapulmonary samples is shown in Table 5. Out of 671 pulmonary samples, 659 (98.2%) were sensitive to rifampicin, while resistance was observed in nine (1.3%) samples. Indeterminate results were noted in three (0.4%) pulmonary samples, including two from sputum and one from bronchial alveolar lavage (BAL). Among the 503 extrapulmonary samples, 495 (98.4%) were rifampicin-sensitive, while four (0.8%) samples showed resistance, including two from bone aspirates, one from lymph nodes, and one from pleural fluid. Indeterminate results were reported in four extrapulmonary samples (0.8%).

**Table 5:** Distribution of sensitivity and resistance to rifampicin in study samples (n = 1174)

Samples	Sensitive	Resistant	Indeterminate	Total
Pulmonary	659 (98.2%)	9 (1.3%)	3(0.4%)	671
Extrapulmonary	495 (98.4%)	4 (0.8%)	4 (0.8%)	503

## Discussion

This study aimed to analyze the performance of Xpert® MTB/RIF in detecting tuberculosis in various pulmonary and extrapulmonary samples. The findings of this study demonstrate the significant diagnostic utility of Xpert® MTB/RIF in TB diagnosis.

Pulmonary and extrapulmonary MTB detection

In our study, 1174 samples were analyzed, comprising 671 pulmonary and 503 extrapulmonary samples. Most of the study participants were middle-aged adults (31-60 years), consistent with the findings of Ana Paulade et al., where the mean age of patients was reported to be 42 ± 19.41 years [11]. Among pulmonary samples, sputum samples yielded the highest MTB detection rate at 48.41%, followed by bronchial washing samples at 16.33%. This highlights the efficacy of Xpert® MTB/RIF in detecting *Mycobacterium tuberculosis* (MTB) in respiratory specimens, particularly in those with high bacterial loads.

Extrapulmonary TB posed more significant diagnostic challenges, yet Xpert® MTB/RIF demonstrated robust performance. Lymph node aspirates showed the highest MTB detection rate at 53.3%, followed by bone pus/aspirates at 47.37%. In contrast, breast tissue, synovial fluid, and pericardial tissue samples exhibited no MTB detection, consistent with findings by Mathur RB et al., which reported lower detection rates in these tissues due to the paucibacillary nature of the disease [9].

Utility in smear-negative pulmonary cases

Smear-negative pulmonary TB cases are diagnostically challenging due to their lower bacterial loads. In this study, Xpert® MTB/RIF detected MTB in 16.55% of smear-negative pulmonary samples, with the association found to be statistically significant (p = 0.043). This underscores the utility of Xpert® MTB/RIF in diagnosing paucibacillary TB, ensuring early and accurate treatment initiation. Similar findings were reported by Vishal Chopra et al., where CBNAAT detected MTB in 58% of smear-negative patients, all of whom were rifampicin-sensitive [12]. Additionally, Casela et al. described a diagnostic gain of 59.9% when comparing Xpert® MTB/RIF with smear microscopy under routine conditions [13]. Another study by Stephen et al. demonstrated that Gene Xpert diagnosed 72.5% of smear-negative samples as positive, further emphasizing its diagnostic superiority in such cases [3]. Few other studies have also shown variable results regarding the diagnostic gain in pulmonary and extrapulmonary samples [14-16].

Correlation with clinical symptoms

The association between symptomatology and MTB detection in pulmonary samples was analyzed in this study, revealing a statistically significant trend (p = 0.05). Among pulmonary TB cases, symptoms such as productive cough with sputum or hemoptysis, as well as systemic symptoms like fever and weight loss, were strongly correlated with positive MTB detection. Similar findings were reported by Vishal Chopra et al., where statistically significant clinical features included mucoid sputum production and evening rise in temperature [12]. These findings emphasize the continued relevance of symptom-based evaluations in guiding diagnostic strategies, especially in resource-limited settings where clinical acumen complements molecular testing [17].

Strengths and limitations

The major strength of this study lies in its large sample size, spanning both pulmonary and extrapulmonary cases over five years. The use of Xpert® MTB/RIF provided reliable data on MTB detection and rifampicin resistance, highlighting its utility as a frontline diagnostic tool. Furthermore, the study underscores the versatility of Xpert® MTB/RIF in addressing the diagnostic challenges posed by smear-negative TB and EPTB.

However, the study also had several limitations. Being retrospective in design, it relied on existing records, which lacked detailed information on patients’ prior treatments, associated risk factors, and co-morbidities such as smoking, alcoholism, and chronic obstructive pulmonary disease (COPD). The absence of data on some key populations restricted a more comprehensive analysis. Additionally, cross-verification of Xpert® MTB/RIF results with the gold standard culture test was not performed, which could have strengthened the findings. These limitations highlight the need for future prospective studies to address these gaps and validate the results.

Clinical and public health implications

The year 2022 marked a significant milestone in TB surveillance efforts in India, with a record-high notification of 24.2 lakh cases, representing a 13% increase compared to 2021. This translates to a case notification rate of approximately 172 cases per lakh population, highlighting the intensified efforts under the National Tuberculosis Elimination Program (NTEP) [18]. Despite these strides, TB continues to present challenges in diagnosis and management, especially in extrapulmonary TB (EPTB) cases, which require precise diagnostic tools due to their atypical presentations and lower bacterial loads.

The high detection rates in smear-negative and extrapulmonary samples make it an indispensable tool in comprehensive TB management strategies. As India intensifies its TB elimination efforts, integrating Xpert® MTB/RIF into routine diagnostic workflows can enhance case detection, reduce diagnostic delays, and enable targeted treatment. Additionally, incorporating clinical symptom evaluations alongside molecular diagnostics ensures a holistic approach, especially in settings with limited resources. Addressing diagnostic challenges in EPTB remains crucial for achieving the ambitious targets of TB elimination in India.

## Conclusions

The Xpert® MTB/RIF assay has demonstrated its critical role in the early and precise diagnosis of tuberculosis, especially in pulmonary and extrapulmonary cases. Its rapid turnaround time and ability to detect MTB in both smear-positive and smear-negative cases provide a significant advantage over traditional diagnostic methods. In pulmonary cases, the assay effectively identified MTB in 48.41% of sputum samples and 16.33% of bronchial wash samples, emphasizing its sensitivity in respiratory samples. Among extrapulmonary cases, the assay showed the highest detection rates in lymph node aspirates (53.3%) and bone pus/aspirates (47.37%) while demonstrating limited utility in breast, synovial, and pericardial tissues. These findings underscore the assay's variable sensitivity depending on sample type. The study confirms that Xpert® MTB/RIF is not just a diagnostic tool but a pivotal element in tuberculosis control strategies, providing timely, accurate results that aid in the effective management of this global health challenge.
